# Nutritional Regulation of Gut Barrier Integrity in Weaning Piglets

**DOI:** 10.3390/ani9121045

**Published:** 2019-11-29

**Authors:** Silvia Clotilde Modina, Umberto Polito, Raffaella Rossi, Carlo Corino, Alessia Di Giancamillo

**Affiliations:** 1 Department of Health, Animal Science and Food Safety, Università degli Studi di Milano, Via Celoria, 10, 20133 Milan, Italy; silvia.modina@unimi.it; 2 Department of Veterinary Medicine, Università degli Studi di Milano, Via Celoria, 10, 20133 Milan, Italy; umberto.polito@unimi.it (U.P.); raffaella.rossi@unimi.it (R.R.); carlo.corino@unimi.it (C.C.)

**Keywords:** gastrointestinal tract (GIT), intestine, morphology and physiology, amino acids, phytochemicals, organic acids, weaning, pig

## Abstract

**Simple Summary:**

Weaning is a very stressful period in the piglet’s life in intensive farming: it is a sudden process occurring between three to four weeks of age, when the gastrointestinal tract (GIT) is still immature. The GIT is formed by the epithelial, immune and enteric nervous system which controls epithelial barrier integrity as well as gut functions including the transport of luminal nutrients, water and electrolytes. Early weaning is characterized by a breakdown of these gut functions, an increase in intestinal permeability and the appearance of gastrointestinal functional disorders, which can have long-lasting consequences in the pig’s life. Weaning, therefore, requires the correct level of nutrients, high quality ingredients, and management, which are directed primarily at encouraging rapid feed intake whilst reducing mortality and morbidity. This review describes the organization of the GIT and highlights the interactions between feed components and the morphology and physiology of the epithelial barrier. Novel dietary strategies focused on improving gut health are also discussed, considering the impacts of selected feed ingredients or additives on the GIT such as functional amino acids, phytochemicals and organic acids.

**Abstract:**

Weaning is very stressful for piglets and leads to alterations in the intestinal barrier, a reduction in nutrient absorption and a higher susceptibility to intestinal diseases with heavy economic losses. This review describes the structures involved in the intestinal barrier: the epithelial barrier, immune barrier and the enteric nervous system. Here, new insights into the interactions between feed components and the physiology and morphology of the epithelial barrier are highlighted. Dietary strategies focused on improving gut health are also described including amino acids, phytochemicals and organic acids.

## 1. Introduction

Weaning is a very stressful period in a piglet’s life. In nature it occurs as a gradual process between 10 to 12 weeks of age, which is near to when the gastrointestinal tract (GIT) matures, while on commercial pig farms weaning occurs suddenly between three to four weeks of age [1]. The intestinal structure is thus crucial for the future health and performance of all young animals. Low feed intake immediately after weaning is responsible for gut morphological alterations such as villous atrophy [2], with consequent lower nutrient absorption and reduced energy available. Unfortunately, this phenomenon occurs during a period when growth is crucial, especially since post-weaning body weight is highly correlated to final body weight [3].

Among all the stressors involved in early weaning, maternal separation is one of greatest, however there are also psychosocial stressors including transportation, mixing, fighting and the establishment of a new social hierarchy, or immunological stressors such as vaccination. Moreover, a sudden change in feed composition from milk-based to solid-based feed associated to an immature digestive system may cause a reduction in nutrient digestion and adsorption: this can be due to a poor gastric hydrochloric acid secretion which is not able to produce sufficient amounts or appropriate digestive enzymes in order to deal with new dietary components [3].

Three to four weeks of age is the period of declining passive immunity from the sow’s milk to the piglet which coincides with commercial weaning, thus heightening the difficulties for the piglet [3]. Improving animal health therefore reduces production losses and increases profits in commercial herds. The intestinal mucosa is constantly exposed to a hard luminal environment including bacteria, toxins and pathogens. It is therefore essential that the intestine is well organized with a complete epithelial barrier to guarantee the safety of the underlying structure (i.e., lamina propria), thus ensuring the survival of the animal. In addition to this defensive action [4], the intestinal mucosa needs to ensure the efficient transport of nutrients such as water and electrolytes for growth, and to selectively take feed and microbial antigens from the luminal content in order to facilitate the development of the mucosal immune system. To perform these contrasting functions, the intestinal mucosa is equipped with a highly specialized barrier mechanism. This review focuses on the specific properties of the epithelial barrier and on the nutrients that modulate or enhance the barrier’s mechanisms.

## 2. Gastrointestinal Barrier

**Figure 1:**
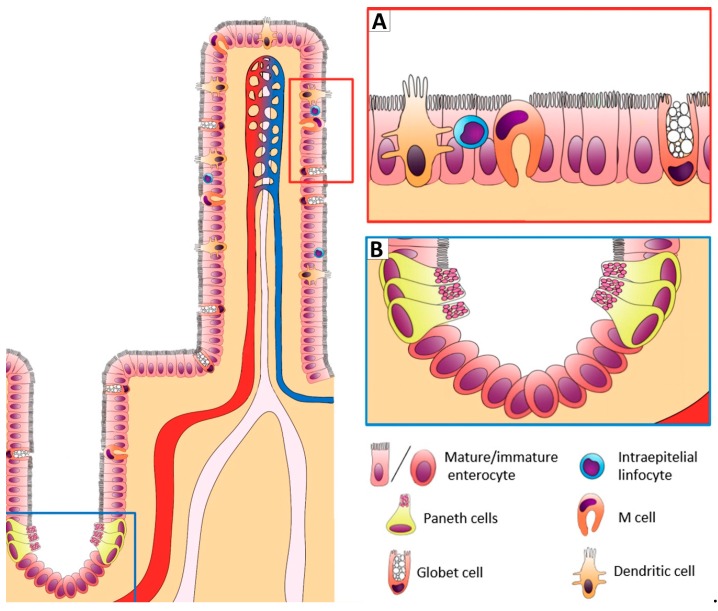
The intestinal epithelial cells of the mucosal lining (enterocytes) and the components of the immune system within the intestinal barrier.

**Figure 2:**
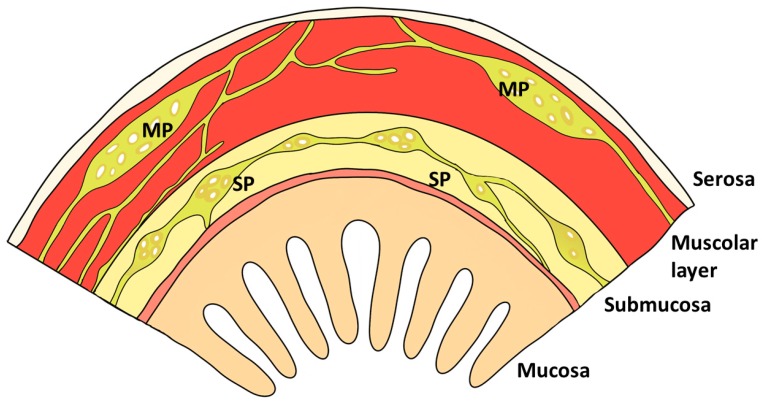
The nervous system that regulates the barrier function. MP: myenteric plexus or Auerbach’s plexus between the longitudinal and circular layers of the muscularis externa; SP: submucosal plexus or Meissner’s plexus in the submucosa.

The GIT barrier is a complex multi-layered organization which acts as a host defense. The structures involved in this barrier are presented in Figure 1 and Figure 2: i) the intestinal epithelial cells of the mucosal lining (enterocytes); ii) the components of the immune system within the intestinal barrier; and iii) the nervous system that regulates the barrier function. A brief overview of the morpho-functional organization of the above-mentioned barrier-components is provided below.

### 2.1. Epithelial Cells

The gastrointestinal tract represents the greatest interface between the external environment and the organism. At the same time, the intestinal mucosa facilitates the digestion and absorption of nutrients through the activity of enzymes and transporters at both the apical and baso-lateral cellular levels of the enterocytes and it is also responsible for the bidirectional transport of water through electrolyte transporters, pumps and channels. Tight junctions (TJ) regulate the permeability of the intestinal barrier. These structures are composed of intracellular and apical inter-cellular membrane proteins e.g., zonula occludens, occludin, and claudins [5,6,7,8]. TJ proteins regulate the “leakiness” of the epithelium through the “gate function”, which selects the epithelial ions and modulates the pore size. TJ proteins also play a critical role in establishing epithelial polarity which maintains the apical Na^+^ gradients necessary for nutrient transport such as glucose, amino acids and water. In addition, as suggested by Turner et al. [9], the epithelium requires a physiological grade of paracellular/transcellular permeability for solute-driven water absorption and transcellular antigen uptake: a mechanism involved in sepsis and multiple organ dysfunction [5,7]. Within the intestinal epithelial barrier, there are also a specialized type of epithelial cells called “goblet cells” (Figure 1), which produce the mucous layer on the intestinal mucosa together with a further cell type, the “Paneth cells”, responsible for the secretion of antimicrobial peptides (Figure 1). Lastly, intestinal crypt epithelial cells secrete Cl^−^ and HCO_3_^−^ ions that provide a physiological buffer for pH regulation, which is an important mechanism against pathogens and endo-luminal stressful conditions [10,11]. The last type of cells belonging to the intestinal barrier are the entero-endocrine cells, which release serotonin and Peptide YY. The latter are neuropeptides that play an important role in pathogen detection as well as in metabolic regulation of the appetite [12,13]. Entero-endocrine cells also express taste receptors [14,15].

### 2.2. Immune System

Intraepithelial lymphocytes (IELs) and M cells (Figure 1A) represent the immune cells within the intestinal barrier. Intraepithelial lymphocytes are composed of a population of T cells [16], which are strategically located among the enterocytes, very close to the luminal milieu, where they induce and regulate immune responses [17,18,19]. The T cells of the intestinal villi are poorly developed in piglets at birth [20,21]. In contrast, they are well represented in postnatal development when exposure to microbials occurs, and it is enhanced after one week or longer (by the critical stage of weaning) [22]. In physiological conditions, IELs also play a fundamental role in the regulation of the turnover of mucosal epithelial cells by eradicating dead/infected cells, thus contributing to epithelial repair/replacement [23]. To date, it is known that correct cellular turnover improves gut health and well-being [24,25,26].

M cells are specialized epithelial cells that differ morphologically and enzymatically from adjacent enterocytes (Figure 1A). M cells act as gatekeepers to the mucosal immune system, continuously sampling the lumen of the small intestine and transporting antigens to the underlying mucosal lymphoid tissue for processing and the initiation of immune responses [27,28,29]. M cell sampling has been exploited to translocate the epithelium by pathogens including *Salmonella typhimurium* [30]. M cells possess a unique intraepithelial invagination or ‘pocket’ (Figure 1), containing B lymphocytes, T lymphocytes, macrophages and dendritic cells which are bone marrow-derived antigen-presenting cells [29]. They are distinguished from intestinal enterocytes by characteristic morphological features. At the apical surface, they have a poorly organized brush border with short irregular microvilli, in contrast to the highly organized brush border of enterocytes, with uniform densely packed microvilli [31]. The usually thick glycocalyx associated with absorptive cells is absent in M cells and is replaced by a thin glycocalyx, which is thought to aid greater access to antigens in the gut lumen. These cells lack certain enterocyte apical surface glycoproteins such as alkaline phosphatase and sucrase–isomaltase, which are typical of the brush-border of enterocytes, and both have been used as negative markers for M cells [32].

### 2.3. Enteric Nervous System

The enteric nervous system (ENS) is quasi autonomous and includes neural circuits that control motor functions, local blood flow, mucosal transport and secretions, and modulate immune and endocrine functions. Specifically, in pigs, the ENS consists of two major neural ganglia located in the muscle (myenteric plexus or Auerbach’s plexus) and in the submucosa (submucosal plexus or Meissner’s plexus), which control motility, peristalsis and mucosal and epithelial functions, respectively, through the constant release of neurochemicals. Auerbach’s plexus is located between the longitudinal and circular layers of the muscularis externa, while Meissner’s plexus is between the longitudinal and circular layers of the muscularis externa (Figure 2) [33].

The nervous system is also a regulator of GIT immune responses via neuro-immune synapses, via the modulation of bacterial toxin detection or adherence [34,35]. Some authors have found that stress neuroendocrine mediators such as catecholamine and the adrenocorticotropic hormone (ACTH) can influence the binding and adherence of pig enteric pathogens to the intestinal mucosa [34,35,36,37,38]. Medland et al. [39] found significant alterations in ENS phenotype and function in response to weaning. Adult pigs that underwent early weaning (16 days of age) showed higher co-localization of intestinal mast cells with enteric nerves, compared with late weaning (28 days of age), which has been correlated with increased severity in functional gastrointestinal disorder symptoms [40].

## 3. GIT Barrier Disorders at Weaning

Many morphological and functional changes occur during weaning in the GIT as reviewed by Witten et al. and Moser et al. [41,42]. These changes induce a failure in the intestinal epithelial barrier characterized by increased permeability [11,43,44], which is much more pronounced if the piglets are weaned too early [45]. The increase in permeability is accompanied by villi modifications [2]: villi height decreases within a minimum of approximately three days after weaning [46,47,48,49,50]. Weaning also negatively affects the proliferation of intestinal crypt epithelial cells [51]. While the epithelial barrier function is altered, a high cytokine production has been reported, revealing the activation of the GIT immune system immediately after weaning [44,52]. Medland et al. demonstrated that early weaning induces a persistent upregulation of the enteric cholinergic system [39], leading to the hypothesis that this may trigger the pathogenic mechanisms that increase disease predisposition related to early life stressors such as weaning [41]. Interestingly, evidence suggests that disorders in the GIT barrier, immune system and nervous system in early weaned pigs remain into adulthood [39,52].

Many nutritional strategies have been adopted to improve gut health and maximize the production of weaned pigs [53,54,55,56]. These strategies have different aims: (1) to improve nutrient digestion and absorption; (2) to regulate gut microbiota in order to obtain a more favorable bacterial species; and (3) to modulate the immune system to enhance disease resistance. In this review, we focus on the impacts of selected additives (functional amino acids, phytochemicals and organic acids), which have trophic effects on the gut barrier of weaned pigs and may alleviate the detrimental effects of weaning on GIT barrier integrity (Table 1). Papers listed in the table refer to the morphological elements that make up the intestinal barrier of weaning piglets (histometry of villi, crypts and their ratio, enterocyte proliferation, goblet cells and epithelial junction). Many other additives have also shown promising results in relation to the health of weaned pigs, but they are not included in the current article.

## 4. Additives

### 4.1. Functional Amino Acids

The functional amino acids glutamine and glutamate are widely used in pig farming. Glutamine and glutamate are the preferred oxidative substrates for intestinal epithelial cells and important sources of carbon atoms for gluconeogenesis. They are thus considered as important fuel for intestinal epithelial cell proliferation and integrity repair [57,58,59]. Glutamate and glutamine are usually classified as non-essential amino acids as they are produced by the body itself [60,61]. However, several studies have led to a redefinition of glutamine as a “conditionally essential” amino acid [62], since, in some instances, autogenous synthesis may be insufficient to meet the body needs, above all during hypermetabolic and stressful periods associated with prolonged starvation, such as weaning [63]. Dietary supplementation with L-glutamine improves growth performance and feed efficiency post weaning by preventing GIT barrier atrophy, increasing villi height in the duodenum and jejunum and in the distal part of the ileum, [53,64,65,66,67] where it induces a decrease in crypt depth, and in the villi:crypt ratio [68]. At the cellular level, the administration of pure glutamine or a mixture of glutamine and glutamate increases the mitosis of enterocytes and reduces the apoptosis of both enterocytes and lymphocytes [53,66]. Glutamine supplementation stimulates innate and adaptive components of immunity, also shown by increased densities of macrophages and IELs [53,66]. All these data corroborate the nutraceutical role of glutamine as a trophic agent for mucosal repair and improvement in barrier function, however it should be taken into account that L-glutamine is expensive and has a low solubility in water and high instability.

To overcome these drawbacks, the synthetic form, alanyl-glutamine (Ala-Gln) has been developed. This dipeptide is highly soluble in water and is more stable and resistant to thermal shock and prolonged storage [68]. Ala-Gln has similar or even higher effects compared to glutamine alone since it can be easily used by the enterocytes, preserving intestinal mucosa integrity and functionality and preventing the development of gastrointestinal disorders resulting from the sudden change from a milk to plant origin diet. This is supported by the higher mucosa thickness in Ala-Gln treated piglets, the increase in villus length and the decrease in crypt depth along the small intestine and the upregulation of the ratio of villi height to crypt depth. This ensures an appropriate area surface of the brush border and nutrient interaction, leading to better digestion and absorption [68].

Ala-Gln association also upregulates the mRNA expression of the epidermal growth factor receptor and the insulin-like growth factor 1 receptor in jejunal mucosa, therefore regulating cell proliferation and differentiation through mitogen-activated protein kinase (MAPK) or phosphatidylinositol-3-kinase (PI3K)/Akt pathways [69]. Ala-Gln prevents intestinal dysfunction and atrophy in weaning piglets by modulating the paracellular trafficking of macromolecules. It has been observed in fact that Ala-Gln dietary supplementation increases the protein levels of occludin and zonulin-1 in the jejunal mucosa and does not affect the protein levels of claudin-1. By contrast, Gln supplementation alone had no effect on the protein levels of occludin, claudin-1, or zonulin-1 in the jejunal mucosa [70]. A reduction has also been described in the jejunal expression of occludin, claudin-1, zonula occludens-2, and zonula occludens-3, but no changes in the abundance of claudin-3, claudin-4, or zonulin-1 in weanling piglets compared with age-matched suckling controls [68]. These results suggest that tight junction proteins may be critical factors that are sensitive to regulation by both weaning stress and glutamine in pigs. Ala-Gln supplementation also influences other mechanisms involved in regulating the GIT epithelial barrier since it increases the number of goblet cells in the duodenal and ileal epithelium, thus enhancing the production of mucins [69] and increasing the protection of intestinal mucosa. This action is very important during weaning, when the mucin level [71] and the goblet cell density decrease [72,73].

Another nutraceutical additive often used in piglet nutrition is the essential amino acid arginine (Arg) [74,75]. Arginine is involved in the synthesis of proteins, urea, polyamine, creatinine, and nitric oxide [76]. Its supplementation in the diet is thus required during periods of maximal growth, injury, and intense stress such as weaning. Arginine deficiency is one of the major factors limiting the growth of young pigs [77]. The intestine plays a key role in Arg absorption, endogenous synthesis and metabolism, as well as in maintaining Arg homoeostasis [78]. Cynober [79] reported that dietary ornithine supplementation, in the form of ornithine alpha-ketoglutarate as an arginine precursor, supports intestinal function by significantly increasing villi height in the duodenum and reducing crypt depth in the jejunum, through increasing polyamine secretion. Similar results were found by Ewtushick et al. [66] who observed that dietary arginine supplementation prevents weaning-induced villous atrophy in the duodenum. Tan et al. [80] also reported that arginine improved DNA synthesis and mitochondrial bioenergetics of intestinal epithelial cells, consequently improving the regeneration and/or repair of the small intestinal mucosa.

Interestingly, the combined action of arginine and glutamine (Arg-Gln) leads to an improvement in the actions of the two amino acids considered alone by preventing villus atrophy and increasing villus height and the villus height:crypt depth ratio, both in the duodenum and jejunum of weaned piglets. This effect is greater than using the single supplementation of either arginine or glutamine [81].

The supplementation of Arg-Gln in the diet increases lactase and sucrose enzyme activities in the enterocytes of the duodenum and the maltase activities in the duodenum and the jejunum. This is of particular importance in the piglet diet, since disaccharidase activities, including sucrose, maltase, and lactase, have long been used as indicators of gut maturity in piglets [82].

### 4.2. Phytochemicals

As mentioned above, weaning impairs intestinal integrity, increases intestinal oxidative stress, and increases the susceptibility of piglets to diseases [83]. The new frontiers used to combat oxidative stress are phytochemical antioxidant compounds in botanical essential oils, i.e., liquid mixtures of volatile compounds obtained from aromatic plants, most commonly by steam distillation.

There are several commonly used phytochemicals (i.e., extracts from oregano, thyme, ginger, fennel, pepper, clove, basil, cinnamon, garlic, mint, etc.) that have strong antioxidant properties in in vitro cell culture as well as in vivo animal models [84]. Phytochemical activities are mainly associated with the antioxidant property of their phenolic compounds which counteract the oxidation of proteins and lipids caused by free radicals. This has been demonstrated by the administration of a plant extract mixture composed of carvacrol, cinnamaldehyde, capsaicin and vitamin E on the oxidative stress induced by a high polyunsaturated fatty acids (PUFA) load in young pigs [85].

To date, limited research has been reported on the effects of phytochemicals on the intestinal oxidative status/responses of weaned pigs. Recent studies have revealed that dietary supplementation of phytochemicals enhanced disease resistance and growth performance [86,87,88]. These benefits are likely driven by improved gut health which maintains normal barrier integrity and function more than its morphology [87,88,89]. The findings on the actions of phytochemicals on gut morphology are still contrasting: dietary supplementation of *E. coli* challenged pigs with capsicum oleoresin, garlic botanical, or turmeric oleoresin has been found, in fact, to alleviate diarrhea and improve the immune response of weaned pigs also at the GIT level without affecting the villi height and crypt depth [87].

On the other hand, oregano promotes intestinal barrier integrity through modulating intestinal bacteria and immune status and these positive effects were mediated by an increase in villus height and the expression of occludin and zonula occludens-1 in the jejunum. [89]. A recent publication by Yuan et al. [90] reported that feeding diquat-challenged pigs with flavones from the leaves of *Eucommia ulmoides* enhanced intestinal morphology by increasing jejunal and ileal villi height and the villous:crypt ratio in diquat-challenged pigs compared with those on a basal diet on day 14. Fiesel et al. [90] found an improved gain:feed ratio in comparison to the control group in pigs fed with polyphenol-rich plant products from grape or hop, but no evidence that this diet supplementation enhanced villus height, crypt depth and their ratio in both the duodenum and jejunum, as well as the apparent total tract digestibility of nutrients. In another study, the same authors reported that polyphenol rich apple pomace or red-wine pomace diets led to an increase in the villus height:crypt depth ratio in the duodenum in weaning piglets [91]. Gessner et al. [92] observed that red-wine pomace had an inhibitory effect on jejunum villi growth, but a stimulating effect on crypt size in the piglet colon and reduced the gut associated lymphoid tissue (GALT) activation via Peyer’s patches in the ileum. Using a mixture of carvacrol, cinnamaldehyde and capsicum oleoresin, Manzanilla et al. [93] found a reduction in the population of intraepithelial lymphocytes (IELs) in the jejunum and ileum, and an increase in lymphocytes in the lamina propria of early-weaned pigs [94].

It is possible that the action of these antioxidants on the intestinal mucosa could be ascribed principally to the immunomodulation of the different cell populations, and that only when used for a second time, they can induce morphological changes in the gut, perhaps also with different timing compared to the various sections of the intestine.

In our research group, we observed that a diet supplemented with verbacoside, a polyphenol plant-derived compound (extract of Verbenaceae leaves, Lippia spp.) had no effect on histo-morphological parameters, however it seemed to have beneficial effects on enterochromaffin cells, which produce serotonin, a pivotal signaling molecule in the brain–gut axis against nitrosamine stress [95]. These data suggest an additional important mechanism of these plant extracts, considering that the pig gut is one of the major sites of serotonin synthesis and release.

Serotonin has a key role in various biological processes in peripheral tissues, such as the regulation of bowel motility and secretion, enterocyte cell proliferation and differentiation, as well as visceral sensitivity [96]. In conclusion, since the effects of these plant origin extracts on the gut environment have not yet been fully clarified, it is important to remember that the properties of the natural extract are subjected to a lot of modifications depending on the method of extraction and on the major active component, and therefore their activity needs to be further investigated [97].

### 4.3. Organic Acids

Organic acids (carboxylic acids) are broadly distributed in nature as elements of plant or animal tissue. They are also produced by the microbial fermentation of carbohydrates, predominantly in the large intestine of pigs. If used as animal feed supplements, with the right doses, they can contribute to increasing bodyweight, improving the feed conversion ratio and to reducing the colonization of pathogens in the intestine [98]. For these reasons, they are frequently used during post-weaning in piglets [99,100,101] when the gut has limited digestive and absorption capacities caused by the insufficient production of hydrochloric acid, pancreatic enzymes and sudden changes in the consistency and intake of feed [102,103]. Indeed, organic acids are able to reduce the luminal pH in proximal GIT by releasing hydrogen ions in the stomach, thereby activating pepsinogen to form pepsin which improves protein digestibility and inhibits GIT Gram-negative indigenous microflora in the gastrointestinal tract. A low pH level in the GIT creates unfavorable conditions for pathogenic bacteria and shows antimicrobial effects: these actions also occur directly in the feed. Organic acids act, directly or indirectly, on the mucosa of the GIT, mostly on the mucosa of the large intestine [104,105]. Evidence suggests that a low pH also increases the digestibility of nutrients through the changes in villus height and depth in the small intestines in young piglets.

Fumaric acid, as a readily accessible energy source, seems to have a local trophic effect on the mucosa in the small intestine and to enhance the absorptive surface and capacity due to a faster recovery of the gastro-intestinal epithelial cells after weaning [106]. Blank et al. [107] observed a positive effect on the length of the villi in the ileum and on the depth of the crypts in the caecum in growing pigs when fed with 0.17% sodium butyrate.

A diet supplemented with benzoic acid increases the gut health of piglets by decreasing the digesta pH values, maintaining the microflora balance, promoting the development of small intestinal morphology (increase in villus height and in the villus height:crypt depth ratio in the duodenum and jejunum) [108]. At the same time, it improves the ileal digestibility of total nitrogen by increasing the villous height and it favorably influences bacterial diversity in the caecum [109].

An increase in epithelial cell proliferation, villi height of the jejunum/ileum and villus height:crypt depth ratio of the duodenum were observed in piglets after a gastric infusion of short chain fatty acids (SCFA; acetic, propionic and butyric acids). Short chain fatty acid infusions also decrease the level of pro-apoptotic proteins, the number of goblet cells in the ileum and colon and the relative mRNA expression of Mucin 1 (MUC1) and claudin-1 in the jejunum, and occludin and claudin-1 in the duodenum and ileum [110]. The positive effect on TJ protein expression has been confirmed by in vitro studies on Intestinal Porcine Epithelial Cell line-J2 (IPEC-J2) cultured in increasing doses of butyrate [111].

Taken together, these observations indicate that supplementation with butyrate supports intestinal integrity, improves intestinal morphology and strengthens intestinal barrier function by boosting the expression of TJ proteins according to Grilli et al. [112], who described a general up-regulation of occludin after buyrate administration, mainly in the small intestine. The same authors, however, observed a down-regulation of claudin-1 in the duodenum, jejunum and ileum of weaned pigs fed with butyrate. Such differences could be related to the dissimilar experimental models and administration, or suggest that the mechanisms by which organic acids modulate TJ take place directly and indirectly on gut barrier integrity; however, this requires further study. In fact, Le Gall et al. [113] described a thinner small-intestinal mucosa and a decreased jejunal villous height after sodium butyrate administration to piglets before or after weaning. On the other hand, Biagi et al. [114] found no modifications in intestinal mucosal morphology in piglets receiving sodium butyrate for six weeks after weaning. Similarly, Ferrara et al. [115] found no influence on the morphometry of the mid-jejunum of piglets fed with fumaric acid, lactic acid, capric acid and caprylic acids, but a beneficial effect on the local immunity by increasing the constitutive number of potential effector cells (principally CD3+ cytotoxic T lymphocytes) to combat infectious diseases. No influence on the nutritional performance and a decrease in the jejunum villi height were observed with fumaric acid, an acidifier blend containing medium-chain fatty acids such as capric acid and caprylic acid [116].

## 5. Conclusions

Weaning stress significantly compromises the health status of piglets, and alterations in the intestinal barrier support the use of functional dietary substances in order to preserve the morphology of the intestine. Many studies focus on the effects of nutrients on the zootechnical parameters, however few have studied the mechanisms and morpho-functional effects. As for the morphology, most studies focus on the dimensions of the villi and crypts, which tend to have positive effects. The present review shows that the effects of phyto- as well as organic acids are more inhomogeneous with negative and zero effects on the gut morphology, however positive effects can be deduced from the gut barrier and immune system. This is because the efficiency of each additive depends on the diet itself, the state of health, and the age of the animals. Today it is therefore not possible to recommend a specific additive that has general positive effects on all piglet diets. However, it is necessary to bear in mind that the specific morpho-functional development of the intestine of piglets from birth to weaning is able to calibrate the administration of additives according to the functional moment of the animals.

## Figures and Tables

**Table 1:** Effects of nutrients during weaning on gut morphology, enterocyte proliferation, goblet cells and epithelial junction: references from the literature are shown.

		Morphology			Enterocyte Proliferation	Goblet Cells	Epithelial Junctions
	Effect	Villi	Crypts	Villi:crypt Ratio			
Amino Acids	Positive	53, 64–67, 69, 79, 81	69, 79, 81	67, 81	53, 66, 70, 80	89	69,69
	Null		67				69
	Negative						
Phytochemicals	Positive	88, 89		89, 91			88
	Null	87, 90, 95	87, 90, 95	90, 95			
	Negative	92					
Organic Acids	Positive	107, 108, 109, 110	107	108, 110	106, 110		111, 112
	Null	114, 115	116	115			
	Negative	113, 116				110	110, 112
